# Application of Alkaline Deep Eutectic Solvents as a Green Alternative to the Traditional Extractants for the Isolation of Humic Substances

**DOI:** 10.1021/acsomega.4c03033

**Published:** 2024-05-30

**Authors:** Dominik Nieweś, Kinga Marecka, Marta Huculak-Mączka

**Affiliations:** Department of Engineering and Technology of Chemical Processes, Faculty of Chemistry, Wroclaw University of Science and Technology, Smoluchowskiego 25, 50-372 Wroclaw, Poland

## Abstract

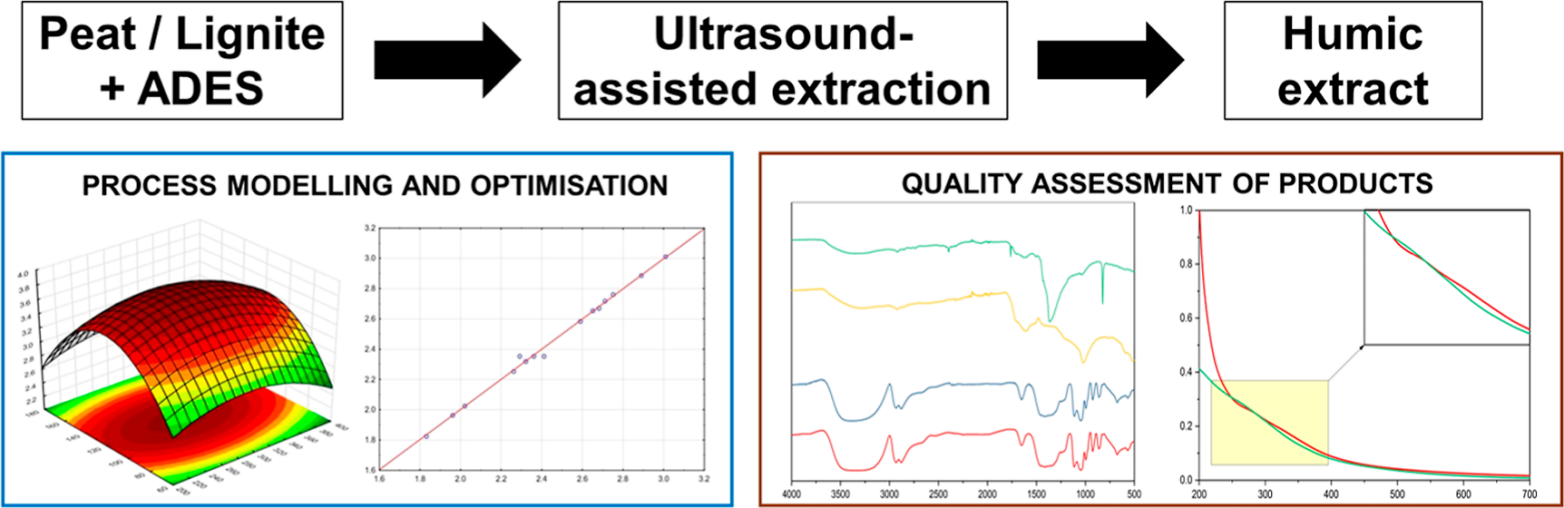

The presented study focused on the possibility of using alkaline deep eutectic solvents (ADESs) as green extractants for the isolation of humic substances (HSs) from peat and lignite in a process intensified by ultrasound. For this purpose, the extraction procedure was statistically described on the basis of the Box–Behnken design, and the carboxyl group content in the obtained products was optimized due to the ADES composition, ultrasound intensity, and extraction time. For optimal extraction conditions, the experimental carboxyl content in the isolated products was equal to 3.71 and 2.96 mmol g^–1^ for the HSs extracted from peat and lignite, respectively. These values were similar to the results for the reference samples, which were HSs extracted using 0.1 M NaOH, as well as humic acids and sodium humates purchased from Sigma-Aldrich. The qualitative assessment of the products obtained was based on spectroscopic methods, including FTIR, ^1^H NMR, and UV–vis. The analyses carried out for the isolated samples revealed the characteristic structures of HSs, including components of aliphatic chains and aromatic core as well as carboxyl, ester, and amino groups. Simultaneously, the results of the spectral ratio of *E*_280_/*E*_472_ showed the significant differences between the relative amount of lignin for the samples tested.

## Introduction

Humic substances (HSs) are defined as macromolecular compounds that are a key source of organic matter, and their presence in the soil profile is crucial to ensure its proper structure.^1,2^ Furthermore, due to their specific molecular structure, HSs have the ability to interact with metal ions; therefore, they can be considered as micronutrient carriers but also as substances for heavy metal immobilization.^3,4^ The mentioned functions determine the main application of HSs for agricultural purposes. Therefore, the use of HSs as fertilizer components, as well as agents for soil bioremediation, has been widely discussed in the literature.^5−8^ However, in recent years, the ideas of alternative applications of HSs, such as food additives, surfactants, and cosmetics, have become evident.^9−12^ The growing use of HSs can be observed on the basis of data in market reports. According to the information given on the Global Market Insights Web site, the size of the market related to only humic acids, which are one fraction of HSs, in 2021 was valued at 532.7 million dollars, when, according to predictions, this value will rise to about 1.1 billion dollars in 2028.^13^

The increase in interest in HSs has led to the search for new technologies for their isolation including unconventional extraction techniques. Traditionally, according to the procedures recommended by the International Humic Substances Society (IHSS) and described in ISO standards, for the isolation of HSs, 0.1 M NaOH is used as the extractant, resulting in low extraction efficiency.^14^ Thus, in recent years, novel techniques that guarantee a higher yield of HS extraction from various raw materials have been tested. The application of hydrothermal extraction, as well as processes assisted by ultrasound or microwave, significantly improved the isolation efficiency, without a negative impact on the molecular structure of HSs.^15,16^ In addition to such process modification, which leads to an increase in the amount of HSs obtained in relation to the mass of raw material used, the application of new types of extractants, including environmentally friendly agents, seems to be a challenge for the extraction technology of HSs. The solution in this regard may be the implementation of deep eutectic solvents (DESs), which are described in the literature as a green alternative to the traditional extractants used. Among the advantages of using deep eutectic extractants, their lower toxicity and higher biodegradation, compared to traditional agents, are mentioned.^17^ Substances of this type have already been extensively tested in bioactive compound isolation processes from waste raw materials.^18,19^ DESs were also used as agents for the processing of chitin, including its isolation, surface modification, and production of chitin-based nanomaterials.^20^ In the context of DES application as extractants, results published by Suopajärvi et al. can be mentioned as an example. They used an alkaline deep eutectic solvent (ADES) for the delignification of waste biomass, resulting in changes in the structure of the network between the nanofibers obtained.^21^

The application of ADES as a green conditioner for activated waste sludge was proposed by Liu et al. The 3D-EEM fluorescence spectra for the extracellular polymeric substances of the sludge, which was treated using a mixture of K_2_CO_3_ and glycerol, showed a significant reduction in the content of HSs in the products, allowing better dewaterability of the sludge, which was the objective of the study.^22^ However, the results showing a decrease in the content of HSs in the sludge after treatment with ADESs may also suggest that a mixture of glycerol and potassium carbonate may be an effective extractant for the isolation of HSs from solid-state raw materials. This aspect was the objective of our study, in which the possibilities of application of ADESs as extractants for the isolation of HSs from peat and lignite in the process assisted by ultrasound were investigated. For this purpose, mixtures of glycerol and K_2_CO_3_ in different molar ratios were prepared, and the isolation process of HS by its use was statistically described according to the results, which were collected according to the experimental matrix based on the Box–Behnken design (BBD). The tested process was optimized for the maximalization of carboxyl group content in the products obtained because of their crucial role for the interaction with metal cations. Humic extracts were qualitatively evaluated using spectroscopic methods (FTIR, ^1^H NMR, and UV–vis), and the results of some methods were compared with the spectra of HSs isolated using 0.1 M NaOH, which is the traditional alkaline extractant for the extraction of HSs, as well as with commercial humic acids and their sodium salts purchased from Sigma-Aldrich.

## Materials and Methods

### Materials

For the process of HS isolation, peat and lignite obtained from Polish deposits were used as raw materials. They were collected from peatland located at the mouth of the Vistula River and the Bechatów lignite deposit, respectively. ADESs were prepared using glycerol and potassium carbonate, which were obtained from Eurochem (Tarnów, Poland) and Chempur (Piekary Śląskie, Poland), respectively. 0.1 M NaOH, which was used as a solution for the isolation of reference humic samples, was prepared by dissolution of solid-state sodium hydroxide, purchased from POCh Avantor (Gliwice, Poland), in deionized water. The analytical humic acids and their sodium salts that were examined as standard samples were obtained from Sigma-Aldrich (Steinheim, Germany). All of the above substances were of analytical purity.

### ADES Preparation

ADESs were prepared according to a procedure that describes the general method of mixing the hydrogen bond acceptor and the hydrogen bond donor.^23,24^ Extractants for the isolation of HSs from peat and lignite were prepared by mixing glycerol and potassium carbonate in three different molar ratios (5:1, 10:1, and 15:1), which was one of the variables that influence the isolation of HSs. The mixtures were then heated to 80 °C on a magnetic stirrer equipped with a heating plate until homogeneous and clear compositions formed. The ADESs obtained were stored in tightly stoppered bottles made of dark glass.

### Extraction Procedure

The procedure tested for the isolation of HSs was based on the method described by Swift, which is recommended by the International Humic Substances Society (IHSS).^25^ However, to intensify the process and adapt it to a new type of extractant, some modifications have been made. They were involved in the application of the thermal process combined with the use of ultrasound, which allowed for improved mass transfer. It was particularly important for the tested process, where a thick viscous extractant was used. First, air-dried peat and lignite were ground to particles of less than 2 mm. Next, 30 g of raw material was placed in conical flaks and mixed with 300 g of extractant. Ultrasound-assisted extraction was carried out with the use of a thermostatic ultrasonic bath at 80 °C. The intensity of ultrasound and the time of the process, similarly to the molar composition of the extractant, were independent factors used to model and optimize the extraction process and ranged between 200 and 400 mW cm^–2^ and 60 and 180 min, respectively. After the extraction process, the phases were separated by centrifugation (4000 rpm at 35 °C by 15 min), and the solid particles, still suspended in liquid, were next removed by vacuum filtration. The humic extracts thus obtained were protonated by the addition of 6 M HCl and analyzed with the methods described in the section “Humic Extracts Analysis”.

### Process Modeling and Optimization

To describe the influence of the three process parameters tested on the extraction procedure, a series of experiments were performed according to the points described by the BBD. The glycerol to K_2_CO_3_ molar ratio values, ultrasound intensity, and extraction time were determined as *X*_1_, *X*_2_, and *X*_3_, respectively, and were coded at three levels, as presented in Table 1. The minimal values of the independent parameters in the considered experimental space were coded as −1, the middle as 0, and the maximal as 1. All experiments were carried out in random order to minimize the effect of unexplained variabilities (noise).

**Table 1:** Levels and Actual Values of Three Independent Factors Tested

levels	independent factors		
	glycerol-to-K_2_CO_3_ molar ratio (*X*_1_)	ultrasound intensity (*X*_2_), mW cm^–2^	time (*X*_3_), min
–1	5:1	200	60
0	10:1	300	120
1	15:1	400	180

The response in this study was the content of carboxyl groups in the molecular structure of the isolated HSs. This parameter was chosen because of its importance for the properties of HSs, which are related to the interactions of HSs with metal cations. This determines possibilities of the use of HSs for agriculture purposes (as micronutrient carriers) and also for environmental applications, including remediation of soil contaminant with heavy metal ions and wastewater treatment. The COOH content was determined by the potentiometric titration method, which was described in detail in the section “Humic Extract Analysis”.

### Humic Extract Analysis

The analyses of the products obtained in the presented study focused on evaluating the chemical structure of the HSs isolated using ADESs in the process tested. For this reason, a series of analytical procedures were applied, including spectroscopic methods and potentiometric titration. Furthermore, some of the methods mentioned were also performed for HSs extracted using 0.1 M NaOH, which is an extractant recommended by IHSS, and for analytical grade humic acids and sodium humates, which are offered by Sigma-Aldrich. Furthermore, the COOH content was also analyzed for the raw materials that were used in this study. In this case, peat was described as P and lignite as L, respectively.

The carboxyl group content in the chemical structure of the HSs tested was determined using the method described by Schnitzer and Gupta and widely applied to humic samples isolated from various raw materials.^26^ The essence of this method is the substitution of carboxyl functional groups by Ca^2+^ ions from calcium acetate. Samples weighing 80–250 mg were shaken with a 25 cm^3^ 0.5 M calcium acetate solution for 24 h at room temperature. The suspension was filtered using syringe filters (0.7 μm), and the residue was washed three times with deionized water. The liquid phase obtained was potentiometrically titrated with a standard 0.1 M sodium hydroxide solution. For this purpose, an automatic potentiometric titrator (HANNA Instruments, HI932 with a HI 1043B pH electrode) was used. A pH of 9.8 was used as the fixed end point of the titration, and the titrant dosing type was dynamic. For comparison, a blank sample without HSs was also included. The carboxyl group content in mmol·g^–1^ was calculated using eq 1, where *V*_s_ and *V*_b_ represent the volume of sodium hydroxide solution used for the titration of the tested HSs and blank samples (cm^3^), *C*_b_ is the molarity of the sodium hydroxide solution (mol·dm^–3^), and *m* is the weight of the exanimated sample (g).
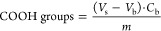
1

For the best possible qualitative description of the humic extracts obtained, three different spectroscopic methods were used. In that part, the humic samples isolated by the use of ADES from peat and lignite, marked as HSP and HSL, were analyzed. The results were compared with the humic samples extracted using 0.1 M NaOH, described as HSP_NaOH_ for the sample isolated from peat and HSL_NaOH_ for the extract obtained from lignite, as well as with the reference humic acids and their sodium salts, purchased from Sigma-Aldrich. They were marked as HA_SIGMA_ and NaH_SIGMA_.

The first method concerned the spectra in the ultraviolet–visible region (UV–vis). The spectral characteristics of the HSs were determined in the wavelength range of 200–800 nm with a scan resolution of 0.2 nm using an ultraviolet–visible spectrophotometer JASCO V-670. HSs were diluted a thousand times with deionized water. The suspensions obtained were then shaken at room temperature for 24 h. The supernatants were filtered by using syringe filters (0.7 μm) and then scanned. Correction was made for a baseline, deionized water.

The presence of various functional groups in the structure of the tested HSs was determined by Fourier transform infrared (FTIR) spectroscopy. Measurements were made for thin films of the tested extracts, which were placed between KBr plates. The spectra were collected at the wavenumber range of 4000–400 cm^–1^ with a resolution of 4 cm^–1^ on a Bruker Vertex 70 spectrophotometer.

The identification of the structures that were characteristic for the humic molecules was also made on the basis of proton nuclear magnetic resonance (^1^H NMR). For each measurement, 100 mg of sample was dissolved in deuterium oxide (D_2_O). The spectra, obtained with 16 scans, were recorded using a Bruker Avance III 500 MHz spectrophotometer at 300 K. The pulse sequence zg30 was applied. The acquisition time and fid resolution were 2.62 s and 0.38 Hz, respectively.

## Results and Discussion

### Statistical Analysis

The concentration of carboxyl groups in the humic extracts obtained under different experimental conditions is presented in Table 2, where the independent factors were given in a coded form. The structure of the BBD matrix with the three independent variables was required for the completion of 15 experiments, and the point that was characteristic for the central of the experimental space, where all independent variables were coded as 0, was analyzed in triplicate. This made it possible to estimate the pure error in the analysis of variance (ANOVA).^27^

**Table 2:** Carboxyl Group Content in HSs Isolated by the Use of ADESs from Peat and Lignite for the Experimental Points According to the BBD for the Three Process Factors Tested

run	independent variables			carboxyl group content in HSs, mmol·g^–1^	
	glycerol-to-K_2_CO_3_ molar ratio (*X*_1_)	ultrasound intensity (*X*_2_)	time (*X*_3_)	extraction from peat (*Y*_peat_)	extraction from lignite (*Y*_lignite_)
1	–1	–1	0	3.43	2.32
2	1	–1	0	2.79	2.75
3	–1	1	0	3.19	2.68
4	1	1	0	2.62	3.01
5	–1	0	–1	2.73	1.96
6	1	0	–1	2.09	2.59
7	–1	0	1	2.63	2.71
8	1	0	1	2.11	2.89
9	0	–1	–1	2.67	1.83
10	0	1	–1	2.51	2.02
11	0	–1	1	2.59	2.26
12	0	1	1	2.43	2.65
13	0	0	0	3.75	2.36
14	0	0	0	3.85	2.41
15	0	0	0	3.90	2.29

### Model Regression and Adequacy

The main element of the analysis of the results obtained was to describe the content of –COOH groups in the structure of HSs as a function of the tested conditions for their isolation. For this purpose, the effect estimates for the tested process were calculated and are presented in Tables 3 and 4. Based on the results of the ANOVA for the effects, the significance of the changes in the process parameters evaluated on the response was determined.

**Table 3:** ANOVA and Effect Estimates of the Calculated Quadratic Model for the HS Extraction from Peat

source	effect	standard error	sum of squares	degree of freedom	mean square	*F*-value	*p*-value	remarks
***X***_**1**_	**–0.59**	**0.04**	**0.70**	**1**	**0.70**	**120.36**	**0**	**significant**
***X***_**2**_	**–0.18**	**0.04**	**0.07**	**1**	**0.07**	**11.41**	**0.01**	**significant**
*X*_3_	–0.06	0.04	0.01	1	0.01	1.24	0.17	not significant
***X***_**1**_^**2**^	**0.49**	**0.03**	**0.90**	**1**	**0.90**	**153.79**	**0**	**significant**
***X***_**2**_^**2**^	**0.33**	**0.03**	**0.41**	**1**	**0.41**	**70.15**	**0**	**significant**
***X***_**3**_^**2**^	**0.95**	**0.03**	**3.34**	**1**	**3.34**	**571.75**	**0**	**significant**
*X*_1_·*X*_2_	0.04	0.05	0.01	1	0.01	0.21	0.53	not significant
*X*_1_·*X*_3_	0.06	0.05	0.01	1	0.01	0.62	0.31	not significant
*X*_2_·*X*_3_	0	0.05	0	1	0	0	1.00	not significant
regression			5.450	5	1.090	363.333		
residual			0.014	5	0.003			
lack of fit			0.002	3	0.001	0.121	0.939	
pure error			0.012	2	0.006			

**Table 4:** ANOVA and Effect Estimates of the Calculated Quadratic Model for the HS Extraction from Lignite

source	effect	standard error	sum of squares	degree of freedom	mean square	*F*-value	*p*-value	remarks
***X***_**1**_	**0.39**	**0.04**	**0.31**	**1**	**0.31**	**84.80**	**0.01**	**significant**
***X***_**2**_	**0.30**	**0.04**	**0.18**	**1**	**0.18**	**49.54**	**0.02**	**significant**
***X***_**3**_	**0.53**	**0.04**	**0.56**	**1**	**0.56**	**153.17**	**0.01**	**significant**
***X***_**1**_^**2**^	**–0.34**	**0.03**	**0.43**	**1**	**0.43**	**118.92**	**0.01**	**significant**
*X*_2_^2^	0.01	0.03	0	1	0	0.03	0.88	not significant
***X***_**3**_^**2**^	**0.16**	**0.03**	**0.09**	**1**	**0.09**	**25.34**	**0.04**	**significant**
*X*_1_·*X*_2_	–0.05	0.06	0.01	1	0.01	0.69	0.49	not significant
***X***_**1**_**·*X***_**3**_	**–0.23**	**0.06**	**0.05**	**1**	**0.05**	**13.94**	**0.04**	**significant**
*X*_2_·*X*_3_	0.10	0.06	0.01	1	0.01	2.75	0.24	not significant
regression			1.640	6	0.273	136.500		
residual			0.008	5	0.002			
lack of fit			0.001	3	0.001	0.048	0.983	
pure error			0.007	2	0.004			

The models for the extraction of HSs from peat and lignite are described in eqs 2 and 3, respectively. Given that polynomials refer to the coded forms of process parameters, only statistically significant effects were considered.
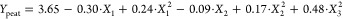
2
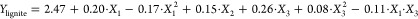
3

**Figure 1:**
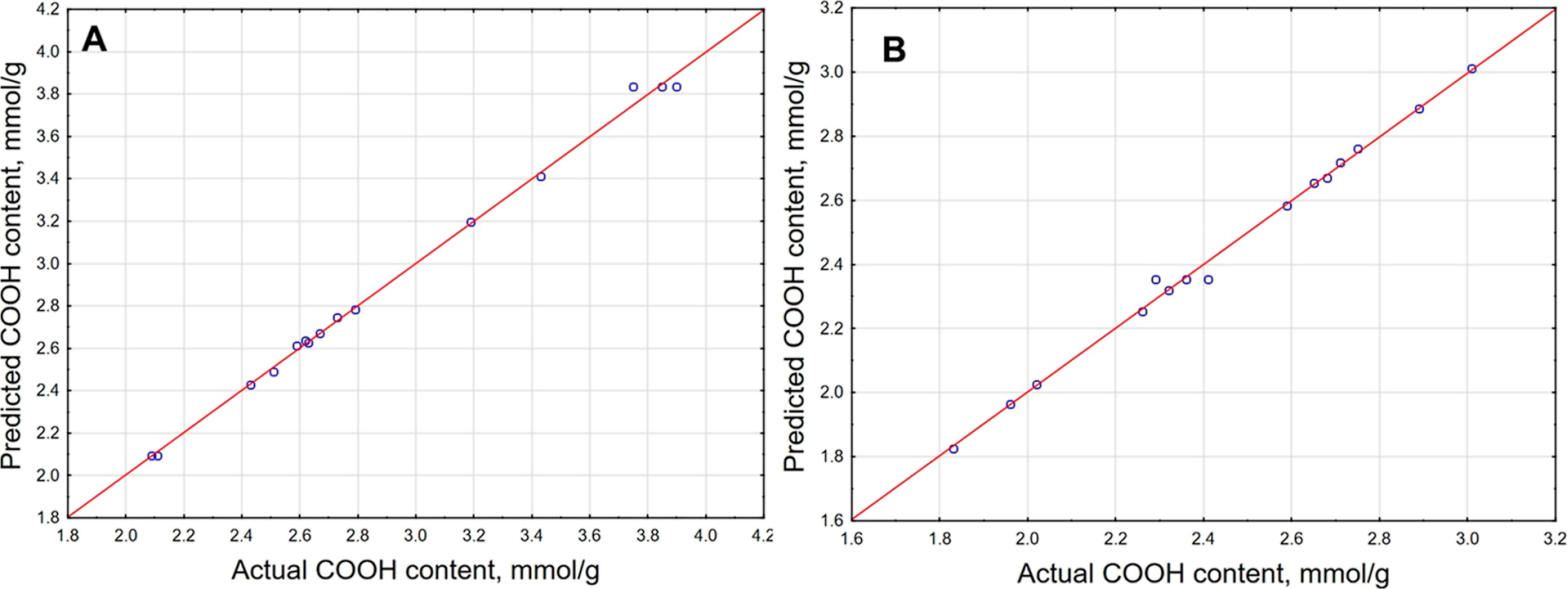
Plots of actual vs predicted results for the extraction of HSs by the use of ADESs as an extractant from peat (A) and lignite (B).

The adequacy of the models presented was evaluated on the basis of the ANOVA. The *p*-values for the lack of fit were equal to 0.939 and 0.983 for eqs 2 and 3, respectively. They were higher than 0.05 for both cases, which means that the statement about the lack of fit for the presented models may be refuted. Furthermore, based on the Fischer test, the calculated *F*-values (*F*_cal._) equaled 363.333 for the model that described the extraction of peat and 136.500 for the equation related to the isolation of HSs from lignite. Comparing the *F*_cal_. values with the tabulated *F*-value (*F*_tab._), which for the designed Box–Behnken matrix was 4.77, may conclude that the presented models describe the experimental results adequately. The well fitting of eqs 1 and 2 was also confirmed by comparing the experimental results with the predictions, which are presented in Figure 1. The coefficients of determination (*R*^2^) were 99.48% for the polynomial that described the extraction of HSs from peat and 98.78% for the equation, which refers to the process where lignite was used as the raw material. This means that only 0.52% of the variances for the isolation of HSs from peat and 1.22% of the variances that described the influence of tested parameters on the efficiency of the extraction of HSs from lignite cannot be explained by the proposed models. The regression values for the relationship between the actual and predicted results (Figure 1) were 99.22 and 98.70% for the models that describe the isolation efficiency of HSs from peat and lignite, respectively.

### Response Surface Analysis and Process Optimization

**Figure 2:**
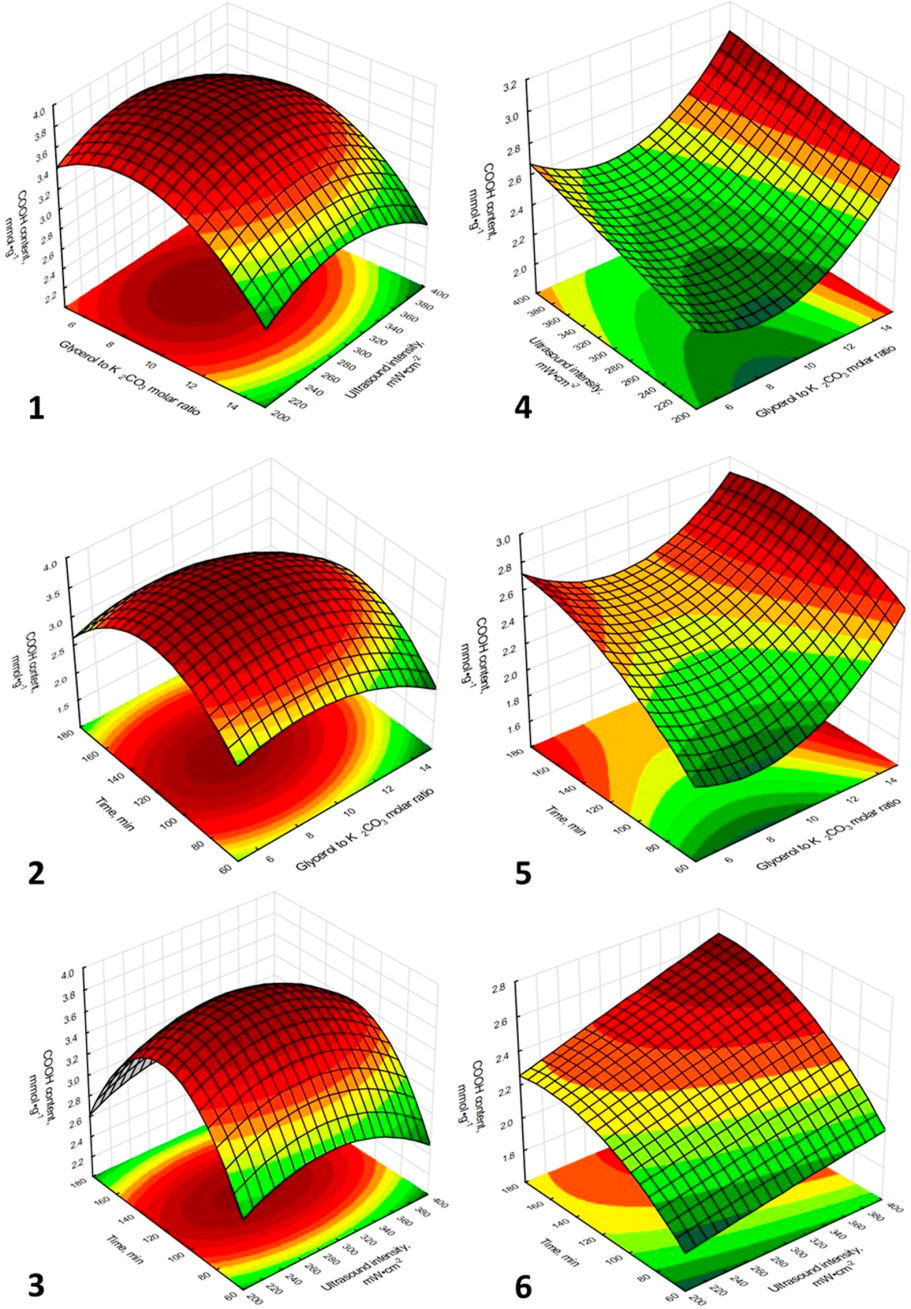
Response surface plots of the influence of tested process parameters on the carboxyl group content in HSs for the extraction with the use of peat (1–3) and lignite (4–6).

The detailed influence of the tested process parameters on the content of carboxyl groups in isolated HSs may be accessed based on the shape of the response surface plots, which describes the response as a function of independent variables (Figure 2). Each of the presented three-dimensional graph describes the influence of two of three variables tested on the COOH content. The value of the third parameter, which was not included in the given plot, was defined as a constant, coded in the experimental matrix as 0.

Comparing the plots, which describe the influence of the same process parameters on the response for the isolation of HSs from peat and lignite (1 vs 4, 2 vs 5, and 3 vs 6), the differences between them can be observed. This is linked to the differences in effect estimates for the tested process, depending on the raw material used. For the extraction of HSs from peat, negative linear effects were observed for three parameters tested, but only linear influences of extractant composition (*X*_1_) and ultrasound intensity (*X*_2_) were statistically significant. Nevertheless, the shapes of graphs 1, 2, and 3 were mainly determined by quadratic effects. The influence of the process parameters on the response for the isolation of HSs from lignite was determined mainly through linear effects, which may be confirmed by the shape of graphs 4, 5, and 6. However, for this process, the significance of the quadratic effects of the glycerol-to-potassium carbonate molar ratio (*X*_1_^2^) and extraction time (*X*_3_^2^) was also observed, the first of which was negative and the second had a positive value. The differences between the statistical results for the processes tested also resulted from the importance of the interaction effects. For the process, where the HSs were isolated from peat, the lack of significance of the interaction effects was observed, while the linear interaction between the extractant composition and the process time (*X*_1_·*X*_3_) had a negative effect on the COOH content in the chemical structure of the HSs isolated from lignite.

Based on the results of the statistical analysis for the processes evaluated, especially using the polynomial models (2) and (3), the optimized parameters of the extraction process were determined for the maximalization of the carboxyl group content in the isolated HSs (Table 5). For the two raw materials tested, differences in the optimal time (*X*_3_) and ultrasound intensity (*X*_2_) may be observed, while the composition of the extractant (*X*_1_) was practically the same. The discrepancies between the process parameters, which were coded as *X*_2_ and *X*_3_, possibly result from the differences in the content of humic fractions in the evaluated raw materials as well as differences in the chemical structure of HSs derived from different sources. Peat is a raw material characterized by a lower degree of carbonization, compared to lignite, and therefore is a richer source of HSs. Moreover, the chemical composition of peat-derived HSs is characterized by a higher proportion of heteroatoms, which affects the increase in the proportion of functional groups, including COOH, in their molecular structure.^28,29^

**Table 5:** Optimal Conditions of the Tested Process for the Maximalization of the COOH Content in Isolated HSs

raw material type	glycerol-to-K_2_CO_3_ molar ratio (*X*_1_)	ultrasound intensity (*X*_2_), mW cm^–2^	time (*X*_3_), min
peat	8.5:1	286	119
lignite	8.6:1	400	170

The calculated COOH content for the optimal extraction parameters was 3.78 and 2.84 mmol·g^–1^ for the isolation of HSs from peat and lignite, respectively. For comparison of predicted optimal responses with experimental results, HSs were isolated from peat (HSP) and lignite (HSL) under the optimal conditions, and the concentration of carboxyl groups for them is presented in Table 6. The concentrations of carboxyl groups for peat (P) and lignite (L) were also presented. The COOH content of the obtained samples was compared with the results for the HSs, which were isolated by using 0.1 M NaOH as an extractant, also from peat (HSP_NaOH_) and lignite (HSL_NaOH_), as well as with commercial humic acids (HA_SIGMA_) and sodium humates (NaH_SIGMA_) purchased from Sigma-Aldrich.

**Table 6:** Carboxyl Group Contents for the Samples Tested

sample	P	L	HSP	HSL	HSP_NaOH_	HSL_NaOH_	HA_SIGMA_	NaH_SIGMA_
COOH content, mmol·g^–1^	1.02	0.88	3.71	2.96	2.87	2.65	3.51	1.23

The results obtained for the compared samples clearly indicate the higher concentrations of carboxyl groups for the humic samples in contrast to peat and lignite, which is due to the presence of contaminants (e.g., mineral residues) in the raw materials. Among the humic samples evaluated, the highest carboxyl group content was observed for commercial humic acids and HSs isolated from peat using the ADES extractant. This is probably due to the type of raw material that was used for the isolation of these samples. Peat is referred to as a source of HSs with a lower degree of humification, resulting in greater participation of heteroatoms and reduction of aromatic structures, compared to HSs isolated from lignite. It also results in the higher amount of oxygen-containing functional groups, e.g., COOH.^30,31^

### Spectroscopic Analysis

The quality assessment of the obtained humic extracts, based on spectroscopic methods, allowed a description of the influence of ADESs and the type of raw material used on the chemical structure of isolated products. In this case, the FTIR, ^1^H NMR, and UV–vis spectra for the humic extracts that were isolated by using an ADES from peat (HSP) and lignite (HSL) were presented. The samples were isolated under optimal process conditions (Table 5). Furthermore, to identify molecular structures, which are characteristic of HSs, in extracts isolated with the use of ADESs, the FTIR spectra obtained were compared with the results for the reference samples, which were HSs extracted using 0.1 M NaOH (HSP_NaOH_ and HSL_NaOH_). In this section, the UV–vis spectra of commercial humic acids (HA_SIGMA_) and their sodium salts (NaH_SIGMA_) obtained from Sigma-Aldrich are also presented.

**Figure 3:**
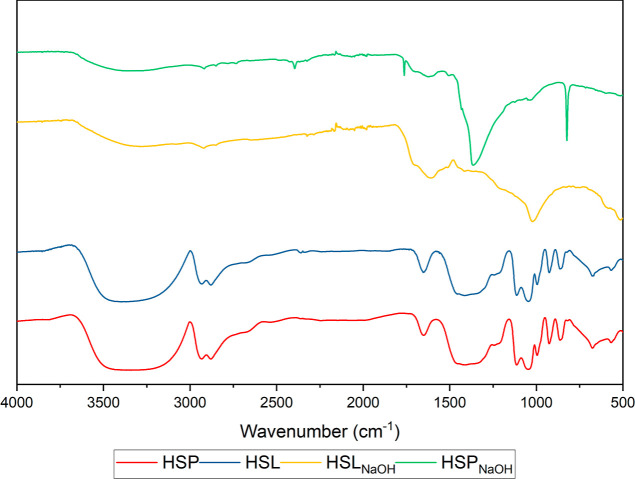
FTIR spectra for the humic samples isolated from peat and lignite by the use of ADES, as well as for the samples extracted using NaOH solution.

The FTIR spectra for the HSs are shown in Figure 3. The broad band at 3600–3200 cm^–1^ corresponds to phenolic groups, but also –OH for hygroscopic water.^32^ The signal related to the vibrations of =CH and =CH_2_ in aromatic rings was observed as a shoulder at 3150–3000 cm^–1^, and the low intensity peaks observed at 2950 and 2850 cm^–1^ can be assigned to the stretching of –CH_3_ and –CH_2_– in aliphatic chains, respectively.^33−35^ The intensive signals at about 1650, 1420, and 1220 were associated with the vibrations of the carboxyl structures and correspond to the C=O stretches, COO^–^ asymmetric stretching, and the deformation of C–O, respectively.^36−38^ The signal peaking at 1020 cm^–1^ represented the C–O stretching of polysaccharides, and the C–H vibrations in aromatic structures were observed in the 860 cm^–1^ band.^39^ The peaks in the region below 700 cm^–1^ may be attributed to the inorganic constituents of the samples tested.^40^

**Figure 4:**
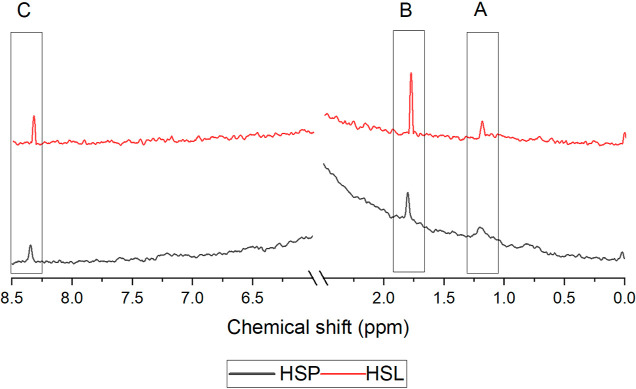
^1^H NMR spectra for HSs isolated from peat (HSP) and lignite (HSL) using ADES as an extractant with the signals for aliphatic structures (A), functional groups (B), and aromatic structures (C).

Analysis of ^1^H NMR spectra for humic extracts was based on the identification of signals in specific resonance areas (Figure 4). In this study, two regions were not taken into account. In the first, between 2.8 and 4.3 ppm, the observed signals may be assigned to alcohols, as well as carbon protons in oxygen-connected methylene groups, which are characteristic of glycerol, being one of the substances used for the preparation of ADES. Therefore, it can be concluded that the peaks in this region corresponded to the extractant that was used for the isolation of HSs. The second area, which was not considered in the analysis of spectra, was the region of 4.3–6.0 ppm, characteristic of the D_2_O shift, which was used as a solvent in the analysis of humic extracts.^41,42^ In the remaining resonance areas, three signals that may be assigned to the molecular structures of HSs were observed. The first one (A), which was peaking below 1.6 ppm, corresponds to the protons of methyl and methylene groups in aliphatic structures.^43^ The second peak (B), between 1.6 and 3.2 ppm, was attributed to the chemical shift of the carboxyl and carbonyl groups and protons of esters and amines.^44,45^ Finally, the signal peaking in the resonance area between 6 and 8.5 ppm (C) can be assigned to the protons of aromatic structures, including quinones and phenols, as well as to the protons of heteroaromatics containing oxygen and nitrogen.^46^

**Figure 5:**
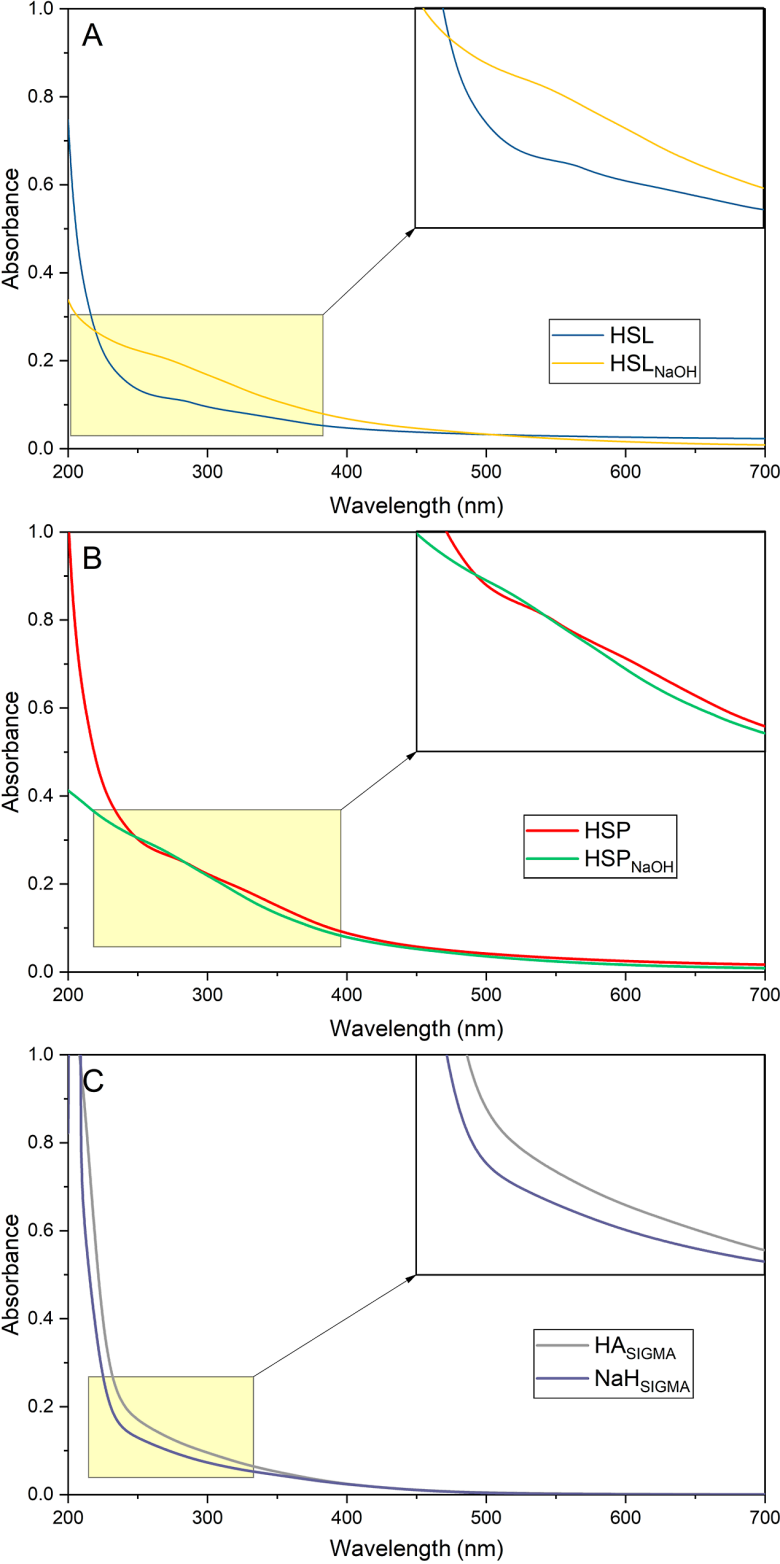
UV–vis spectra for the humic samples isolated from lignite (A) and peat (B) by the use of ADES and 0.1 M NaOH, as well as for the reference samples purchased from Sigma-Aldrich (C).

The UV–vis analysis allowed us to determine the chemical structure of HSs in the context of their degree of humification. For this purpose, in addition to the spectra presented in Figure 5, the characteristic absorbance ratios were calculated for the tested samples (Table 7). The UV–vis spectra revealed the differences that mainly resulted from the presence of various humic fractions in the tested samples as well as the type of raw material from which the HSs were isolated. Generally, for all tested samples, the absorbance values decreased with an increasing wavelength. However, in Figure 5A,B, which presented, respectively, the spectra of HSs isolated from lignite and peat, the characteristic inflection was observed at approximately 280 nm. This signal is characteristic for the C=O chromophores of the fulvic fraction.^47^ It corresponds to the composition of these samples. In the case of humic extracts, whose spectra are presented in Figure 5A,B, the fraction of humates and humic acids was not separated by lowering the pH of the liquid phase after extraction, and therefore, the fulvic fraction was presented in these samples. Upon comparison of the spectra because of the type of raw material from which the HSs were obtained, it can be concluded that the signal mentioned is more intense for the samples, which were isolated from peat. This is related to the differences in the degree of humification of the raw materials used. Peat, compared to lignite, is characterized by a higher amount of humic fractions with a lower degree of condensation, also resulting in a higher proportion of fulvic fractions in isolated HSs.^48,49^

**Table 7:** Spectral Ratios for the HSs Evaluated

sample	HSP	HSL	HSP_NaOH_	HSL_NaOH_	HA_SIGMA_	NaH_SIGMA_
*E*_665_/*E*_465_	0.36	0.55	0.34	0.58	0.64	0.67
*E*_280_/*E*_472_	3.84	3.15	12.89	12.64	4.81	5.06
*E*_365_/*E*_250_	0.22	0.25	0.26	0.29	0.42	0.48
*E*_270_/*E*_400_	4.02	3.35	3.21	2.85	2.66	2.43

The spectral ratios for the tested humic samples were calculated and interpreted according to Sarlaki et al. and Boguta et al.^50,51^ The indexes given in Table 7 refer to the wavelengths at which the absorbance data were collected. Generally, due to the information provided, the values of the determined ratios were interpreted in two groups. The first, which included the results of *E*_665_/*E*_465_ and *E*_280_/*E*_472_, referred to the transformation of samples, and the mentioned ratios determined the degree of humification and the relative amount of lignin, respectively. The second part of the analysis was associated with the chemical structure of the HSs. It was based on the values of the *E*_365_/*E*_250_ and *E*_270_/*E*_400_ ratios, which were appropriately related to the molecular weight, structural condensation, and carboxylic compound content in the molecular structure of the samples tested.

Analyzing the values of the spectral ratio that was connected to the degree of humification, it can be concluded that this parameter for the humic samples tested mainly depended on the type of the raw material, from which the HSs were isolated, and the type of extractant used had no significance effect. The value of the *E*_665_/*E*_465_ ratio was higher for the humic samples extracted from lignite. This means that these samples were characterized by a higher degree of humification, which is associated with the differences in the conversion of organic matter in peat and lignite. The *E*_665_/*E*_465_ results for commercial samples were higher than those, which was characteristic of the HSs isolated in this study. It is caused by the fact that the Sigma-Aldrich samples contained only humic acids (HA_SIGMA_) and humates (NaH_SIGMA_), while the humic extracts, which were isolated by the use of ADES and NaOH, in addition to the humic acids and their salts, also included the fulvic fraction. Evaluation of the relative amount of lignin (*E*_280_/*E*_472_ ratio) in the isolated samples allowed the conclusion that the application of ADESs resulted in the isolation of extracts with a lower lignin content, especially compared to the use of 0.1 M NaOH as an extractant. The mentioned differences may be caused by the partial depolymerization of lignin, which was described by Yue et al., who observed this process during the treatment of waste lignin using a mixture of glycerol/K_2_CO_3_, under similar conditions, which were applied in this study for isolation of HSs.^52^

The results of the *E*_365_/*E*_250_ ratio for the evaluated samples indicate that the molecular size and degree of condensation of HSs depended mainly on the type of raw material from which the HSs were isolated, which is consistent with the conclusions about the degree of humification and points to the correlation between the degree of organic matter conversion and the molecular structure of the HSs, which were isolated from a given raw material.^53−55^ Based on the comparison of the *E*_365_/*E*_250_ values for the isolated humic extracts (HSP, HSL, HSP_NaOH_, and HSL_NaOH_) with the samples purchased from Sigma-Aldrich (HA_SIGMA_ and NaH_SIGMA_), it can be observed that the HSs obtained in this study were characterized by lower condensation, which is caused by the presence of fulvic substances in the humic extracts, which are a fraction with a lower degree of condensation and molecular weight, compared to humic acids and humates.^56,57^ The values of the last absorbance ratio determined in this study (*E*_270_/*E*_400_) for the samples analyzed indicated a higher carboxyl content in the samples extracted using ADESs. When comparing the results by raw material type, a higher value of *E*_270_/*E*_400_ was observed for HSs extracted from peat. This is associated with a higher proportion of functional groups containing oxygen, including COOH. The lower carboxyl content in commercial Sigma-Aldrich samples can be explained by the fact that these samples contained only the humic fraction, while the HSs extracted in this study also had fulvic substances in their composition, which are defined as a fraction with a higher content of functional groups.^58^

## Conclusions

The molecular structure and the resulting properties that affect the improvement of soil structure and fertilization efficiency determine the use of HSs (HSs) for agricultural purposes. However, new possibilities of using these macromolecular compounds are noticed. In this study, the application of ADESs for the isolation of HSs from peat and lignite was evaluated.

The conditions of the ultrasound-assisted process were optimized on the basis of the BBD to maximize the COOH content in the extracts obtained. The effect estimates of the independent parameters tested on the carboxyl group content showed differences in significance depending on the type of the raw material, which were reflected in the shape of the response surface plots. Generally, for the process where the HSs were isolated from the peat, significant negative linear effects of the extractant composition and ultrasound intensity were observed, whereas the quadratic effects of the parameters mentioned were positive. In the case of using lignite as a raw material, among the significant sources, all linear effects and the quadratic effect of time were positive, while the quadratic effect of extractant composition and interaction between the glycerol/K_2_CO_3_ molar ratio and time were negative. The statistical analysis of the results, including the *p*-values for the lack of fit that were higher than 0.05, and the coefficients of determination (*R*^2^) of about 99%, highlights their significance of the created models. Furthermore, the regression values for the relationship between the actual and predicted results for the models that describe the isolation efficiency of the HSs were also approximately 99% for both raw materials tested. It was confirmed by experimental verification of the process under the optimal conditions determined, which showed minimal differences between the actual results and the predicted COOH content for HSs isolated from peat and lignite by using glycerol/K_2_CO_3_ mixtures.

The ^1^H NMR analysis of the extracts revealed the presence of signals in three defined resonance areas that can be assigned to the characteristic structures of HSs including aliphatic chains (below 1.6 ppm), functional groups (1.6–3.2 ppm), and aromatic structures (6–8.5 ppm). It was also confirmed by the comparison of FTIR results for samples that were isolated by the use of ADESs, with the spectra for the HSs extracted by the use of 0.1 M NaOH, which revealed that the presence of peaks corresponds to the vibration of aliphatic chains, aromatic rings, and polysaccharide structures. Moreover, the UV–vis spectra showed an inflection at approximately 280 nm, which may be attributed to the chromophores of the fulvic fraction. The comparison of spectral ratio results for HSs extracted using ADESs, NaOH solution, and for commercial samples showed the differences in the degree of condensation and humification, which results mainly from the type of the raw material used and the presence of the fulvic fraction in the extracted HSs, which the humic acids and sodium humates purchased from Sigma-Aldrich did not include. Especially interesting is the significant difference in the *E*_280_/*E*_472_ results for the samples isolated using ADESs and 0.1 M NaOH, indicating a different relative lignin content. The reason for this fact may be the partial depolymerization of lignin in the raw material under the influence of the glycerol/K_2_CO_3_ mixture.

In summary, the results presented show the effectiveness of application of ADESs based on glycerol and potassium carbonate for the isolation of HSs with significant carboxyl content in their molecular structure. Thus, the application of glycerol-based extractants for the isolation of HSs may be the basis for the implementation of new types of solvents for this process, including mixtures based on waste glycerol, which may represent a new approach for the isolation of HSs in the context of circular economy, based not only on innovations in the application of waste raw materials (e.g., compost) but also on the use of waste-derived substances for the extractant preparation.
